# LDPC Coded Massive MIMO Systems

**DOI:** 10.3390/e21030231

**Published:** 2019-02-27

**Authors:** Inho Hwang, Han Jin Park, Jeong Woo Lee

**Affiliations:** School of Electrical and Electronics Engineering, Chung-Ang University, 84 Heukseok-ro, Dongjak-gu, Seoul 06974, Korea

**Keywords:** massive MIMO, LDPC codes, joint detection and decoding, low complexity, density evolution

## Abstract

We design a coded massive multiple-input multiple-output (MIMO) system using low-density parity-check (LDPC) codes and iterative joint detection and decoding (JDD) algorithm employing a low complexity detection. We introduce the factor graph representation of the LDPC coded massive MIMO system, based on which the message updating rule in the JDD is defined. We devise a tool for analyzing extrinsic information transfer (EXIT) characteristics of messages flowing in the JDD and the three-dimensional (3-D) EXIT chart provides a visualization of the JDD behavior. Based on the proposed 3-D EXIT analysis, we design jointly the degree distribution of irregular LDPC codes and the JDD strategy for the coded massive MIMO system. The JDD strategy was determined to achieve a higher error correction capability with a given amount of computational complexity. It was observed that the coded massive MIMO system equipped with the proposed LDPC codes and the proposed JDD strategy has lower bit error rate than conventional LDPC coded massive MIMO systems.

## 1. Introduction

The massive multiple-input multiple-output (MIMO) system, whose transmitter and receiver are equipped with tens to hundreds of antennas, has recently attracted many researchers and engineers because it can vastly improve the transmission data rate and spectral efficiency [1,2,3,4,5,6,7]. Massive MIMO technology has recently found successful applications in cellular networks, known as the fifth generation (5G) systems [5,6,7,8,9,10,11], as well as in energy-efficient wireless sensor networks [12,13,14,15].

Recovering multiplexed data from signals received by many antennas in an optimal manner requires tremendously high amount of computations, so the reduction of detection complexity has been a great concern for utilizing the massive MIMO technique in practical communication systems [3,4]. As an approach to reduce the detection complexity, suboptimal linear detection algorithms have been intensively studied [7,16,17,18,19,20,21,22,23,24,25,26], where matched filter (MF) detection, zero forcing (ZF) detection and minimum mean squared error (MMSE) detection are well known examples. Nevertheless, these linear detection schemes cannot lower the computational complexity of the massive MIMO receiver to an acceptable level because the inversion of high dimensional matrices is still required. Then, low complexity detection algorithms based on approximate matrix inversion [18,19], low complexity factor graph (FG) based belief propagation (BP) algorithms [20,21,22] and pairwise Markov random fields (MRF) based MIMO detection algorithms [20,21] have been proposed. Tree-searching soft-input soft-output (SISO) MIMO detection algorithms have also been proposed in various forms [23,24,25,26]. The FG based BP detection with Gaussian approximation of interference (GAI), called FG-GAI BP detector, was proposed as one of the promising solutions to reduce the computational complexity of the massive MIMO receiver to the practically allowable level [21,22].

Low-density parity-check (LDPC) codes have been widely used in various communication systems thanks to the powerful error correction capability [27,28,29]. It is well known that LDPC codes can be efficiently designed by using the density evolution algorithm [29,30] or the extrinsic information transfer (EXIT) chart [31]. There have been many research activities regarding the analysis and design of LDPC coded MIMO systems with various forms of detection and decoding mechanisms [32,33,34,35,36]. It is a natural approach to apply LDPC codes to the massive MIMO system to improve the transmission reliability, where a joint detection and decoding (JDD) algorithm of low complexity, of course, needs to be considered. In [37], non-binary LDPC codes are designed for coded massive MIMO systems considering modified MMSE and MF soft-output detectors. In [22,38], binary LDPC codes and non-binary LDPC codes, respectively, are designed by considering the FG-GAI BP detection algorithm through the degree distribution optimization based on the EXIT chart analysis.

To make the LDPC coded massive MIMO technology more applicable in practical communication systems, the convergence of JDD has to be sped up for a given amount of computational complexity. Note that the faster JDD convergence results in the lower BER if the computational complexity is limited to finite amount. In [39], a modified FG-GAI BP detection algorithm is proposed to improve the convergence rate of JDD in LDPC coded massive MIMO systems at the cost of increased computational complexity. It is notable that one JDD iteration can be composed of multiple detection iterations followed by multiple decoding iterations. Thus, the JDD strategy, specified by the ratio of the number of detection iterations and the number of decoding iterations composing one JDD iteration, can be used as a design parameter to obtain a good trade-off between the error correction performance and the computational complexity. However, there do not exist many research works on developing a systematic way to determine the JDD strategy improving the BER performance with a given amount of computational complexity and an efficient way to design LDPC codes depending on the structure of JDD strategy. Thus, there exist strong needs for a systematic and efficient design procedure of LDPC codes and JDD strategy for coded massive MIMO systems.

In this paper, we design the LDPC coded massive MIMO system with an iterative JDD algorithm, where the suboptimal FG-GAI BP detection is considered. We represent the LDPC coded massive MIMO system by a factor graph composed of observation nodes, middle nodes, variable nodes and check nodes connected through edges, and we define iterative updating rules for messages flowing over the factor graph of JDD. We propose an analysis tool for investigating the EXIT behavior of JDD, by which the density evolution of messages is analyzed and a 3-D (three-dimensional) EXIT chart visualization is obtained. Based on the proposed EXIT analysis, we design jointly irregular LDPC codes and the JDD strategy for the coded massive MIMO system to attain the lowest BER with a given amount of computational complexity. In the LDPC code design, we include an additional constraint regarding the placement of edges between variable nodes and check nodes in a practical point of view. It is observed that the coded massive MIMO system equipped with the proposed LDPC codes and the proposed JDD strategy has a lower BER performance than that equipped with conventional LDPC codes and conventional JDD strategy. The performance gain of the proposed scheme over conventional schemes are noticeable, especially when a low amount of computational complexity is allowed for the receiver of the coded massive MIMO system.

This paper is organized as follows. In Section 2, we present the model for coded massive MIMO system. In Section 3, we introduce the operation of JDD, propose the EXIT analysis tool for JDD, and analyze the EXIT behavior of JDD of the LDPC coded massive MIMO system. In Section 4, we design LDPC codes and the JDD strategy by using the proposed EXIT analysis tool. In Section 5, we present BER performances of the proposed LDPC coded massive MIMO system in various points of view and compare those with conventional ones. Finally, we conclude this paper in Section 6.

### Notations

- $n_{T}$: Number of transmit antennas.
- $n_{R}$: Number of receive antennas.
- $N_{ch}$: Total number of channel uses required to transmit all symbols.
- C: Complex number.
- R: Real number.
- A: Set of values for complex transmit symbols.
- $\bar{A}$: Set of values for real-valued transmit symbols.
- $M_{o}$: Modulation order of complex transmit symbols, where $M_{o}$ is the cardinality of A.
- $x^{\left(l\right)}$: Transmit symbol vector at the *l*th channel use, $l=1,2,\cdots ,N_{ch}$, where $x^{\left(l\right)}\in A^{n_{T}\times 1}$.
- $y^{\left(l\right)}$: Received signal vector at the *l*th channel use, where $y^{\left(l\right)}\in C^{n_{R}\times 1}$.
- $w^{\left(l\right)}$: Additive noise vector at the *l*th channel use, where $w^{\left(l\right)}\in C^{n_{R}\times 1}$.
- $H^{\left(l\right)}$: MIMO channel gain matrix at the *l*th channel use, where $H^{\left(l\right)}\in C^{n_{R}\times n_{T}}$.
- ${\bar{x}}^{\left(l\right)}$, ${\bar{y}}^{\left(l\right)}$, ${\bar{w}}^{\left(l\right)}$, ${\bar{H}}^{\left(l\right)}$: Real-valued representations of $x^{\left(l\right)}$, $y^{\left(l\right)}$, $w^{\left(l\right)}$ and $H^{\left(l\right)}$, respectively, where ${\bar{x}}^{\left(l\right)}\in {\bar{A}}^{2n_{T}\times 1}$, ${\bar{y}}^{\left(l\right)}\in R^{2n_{R}\times 1}$, ${\bar{w}}^{\left(l\right)}\in R^{2n_{R}\times 1}$, ${\bar{H}}^{\left(l\right)}\in R^{2n_{R}\times 2n_{T}}$.
- ${\bar{x}}_{i}^{\left(l\right)}$, ${\bar{y}}_{i}^{\left(l\right)}$, ${\bar{w}}_{i}^{\left(l\right)}$: The *i*th entry of ${\bar{x}}^{\left(l\right)}$, ${\bar{y}}^{\left(l\right)}$, ${\bar{w}}^{\left(l\right)}$, respectively.
- ${\bar{h}}_{ij}^{\left(l\right)}$: The (i,j)th entries of ${\bar{H}}^{\left(l\right)}$.
- ℜ{·}: Real part of a complex value.
- ℑ{·}: Imaginary part of a complex value.
- E{·}: Expectation.
- Var{·}: Variance.
- ${\mathrm{DET}}_{l}$: Detector node corresponding to the *l*th channel use.
- $y_{\setminus i}$: Vector obtained by excluding the *i*th entry of y.
- $N_{g}$: Number of global JDD iterations.
- $N_{det}$: Number of detection iterations in one global iteration.
- $N_{dec}$: Number of decoding iterations in one global iteration.
- I(U;X): Mutual information between *U* and *X*.

## 2. Modeling of LDPC Coded Massive MIMO System

Consider a massive MIMO system with $n_{T}$ transmit antennas and $n_{R}$ receive antennas. A *K*-bit information sequence b is encoded to a *N*-bit LDPC codeword u with the code rate of R=K/N. Then, *N* coded bits are modulated as $M_{o}$-ary QAM symbols to be transmitted by $n_{T}$ transmit antennas using a spatial multiplexing over $N_{ch}$ channel uses. Note that $n_{T}$ symbols are transmitted at each channel use resulting in $N_{ch}=\left\lceil \frac{N}{n_{T}\log_{2}M_{o}}\right\rceil$. The MIMO channel at the *l*th channel use is expressed as

(1) $$y^{\left(l\right)}=H^{\left(l\right)}x^{\left(l\right)}+w^{\left(l\right)},\;l=1,2,\cdots ,N_{ch},$$

where entries of $w^{\left(l\right)}$ are independent and identically distributed (i.i.d.) zero-mean circular symmetric complex white Gaussian with variance of $\sigma^{2}$, and entries of $H^{\left(l\right)}$ are i.i.d. circular symmetric complex Gaussian with zero mean and unit variance. The real-valued representation of Equation (1) is written by

(2) $${\bar{y}}^{\left(l\right)}={\bar{H}}^{\left(l\right)}{\bar{x}}^{\left(l\right)}+{\bar{w}}^{\left(l\right)},$$

where

$$\begin{array}{l} {\bar{y}}^{\left(l\right)}=\left[\begin{array}{c} ℜ\left\{y^{\left(l\right)}\right\}\\ ℑ\left\{y^{\left(l\right)}\right\} \end{array}\right]\in R^{2n_{R}\times 1},\;{\bar{x}}^{\left(l\right)}=\left[\begin{array}{c} ℜ\left\{x^{\left(l\right)}\right\}\\ ℑ\left\{x^{\left(l\right)}\right\} \end{array}\right]\in {\bar{A}}^{2n_{T}\times 1},\\ {\bar{w}}^{\left(l\right)}=\left[\begin{array}{c} ℜ\left\{w^{\left(l\right)}\right\}\\ ℑ\left\{w^{\left(l\right)}\right\} \end{array}\right]\in R^{2n_{R}\times 1},\;{\bar{H}}^{\left(l\right)}=\left[\begin{array}{l} ℜ\left\{H^{\left(l\right)}\right\}\;-ℑ\left\{H^{\left(l\right)}\right\}\\ ℑ\left\{H^{\left(l\right)}\right\}\;ℜ\left\{H^{\left(l\right)}\right\} \end{array}\right]\in R^{2n_{R}\times 2n_{T}}. \end{array}$$

**Figure 1:**
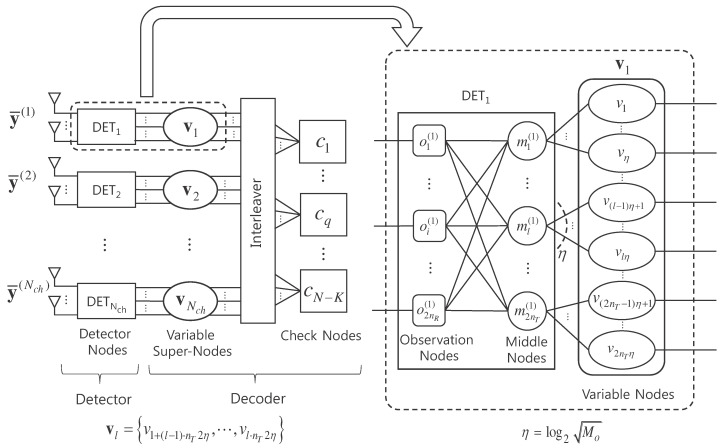
Receiver structure of LDPC coded massive MIMO system.

The receiver of massive MIMO system with the real-valued representation given in Equation (2) can be expressed by a bipartite graph shown in Figure 1. The receiver consists of a detector and a decoder which exchange messages with each other iteratively by joint detection and decoding. The detector is composed of $N_{ch}$ detector nodes, ${\mathrm{DET}}_{l}$, $l=1,\cdots ,N_{ch}$, while the decoder is composed of *N* variable nodes, $v_{1},\cdots ,v_{N}$, and N−K check nodes, $c_{1},\cdots ,c_{N-K}$. Each detector node ${\mathrm{DET}}_{l}$ is composed of $2n_{R}$ observation nodes, $o_{1}^{\left(l\right)},\cdots ,o_{2n_{R}}^{\left(l\right)}$, and $2n_{T}$ middle nodes, $m_{1}^{\left(l\right)},\cdots ,m_{2n_{T}}^{\left(l\right)}$, connected through edges. Each middle node is connected to $\log_{2}\sqrt{M_{o}}$ variable nodes, where each real-valued symbol is generated from $\log_{2}\sqrt{M_{o}}$ bits. We define variable super-nodes, $v_{1},\cdots ,v_{N_{ch}}$, each of which is a group of variable nodes associated with symbols transmitted at each channel use. Signals ${\bar{y}}^{\left(1\right)}$, ⋯, ${\bar{y}}^{\left(N_{ch}\right)}$ received over $N_{ch}$ channel uses are input to detector nodes ${\mathrm{DET}}_{1},\cdots ,{\mathrm{DET}}_{N_{ch}}$, respectively.

## 3. Joint Detection and Decoding for LDPC Coded Massive MIMO System

### 3.1. Operation of Joint Detection and Decoding

We consider an iterative JDD process employing a low-complexity detection algorithm based on FG-GAI BP [22] and a sum-product decoding algorithm. One JDD iteration is composed of $N_{det}$ detection iterations followed by $N_{dec}$ decoding iterations, where we call a JDD iteration as a global iteration. Let us consider the *l*th channel use. Then, Equation (2) can be written as

(3) $${\bar{y}}_{i}^{\left(l\right)}=\sum_{j=1}^{2n_{T}}{\bar{h}}_{ij}^{\left(l\right)}{\bar{x}}_{j}^{\left(l\right)}+{\bar{w}}_{i}^{\left(l\right)},\;i=1,\cdots ,2n_{R}.$$

Each observation node $o_{i}^{\left(l\right)}$ obtains the information of ${\bar{x}}_{k}^{\left(l\right)}$, $k=1,\cdots ,2n_{T}$, from ${\bar{y}}_{i}^{\left(l\right)}$ by regarding terms associated with ${\bar{x}}_{k}^{\left(l\right)}$, j≠k, as interferences. For this purpose, we define $z_{ik}^{\left(l\right)}≜\sum_{j=1,j\neq k}^{2n_{T}}{\bar{h}}_{ij}^{\left(l\right)}{\bar{x}}_{j}^{\left(l\right)}+{\bar{w}}_{i}^{\left(l\right)}$ as the interference plus noise when detecting the symbol ${\bar{x}}_{k}^{\left(l\right)}$ and rewrite Equation (3) as

(4) $${\bar{y}}_{i}^{\left(l\right)}={\bar{h}}_{ik}^{\left(l\right)}{\bar{x}}_{k}^{\left(l\right)}+z_{ik}^{\left(l\right)}.$$

In the case of using a massive number of transmit antennas, we can approximate $z_{ik}^{\left(l\right)}$ as a Gaussian random variable [22] with the mean $\mu_{z_{ik}^{\left(l\right)}}$ and the variance $\sigma_{z_{ik}^{\left(l\right)}}^{2}$, where

(5) $$\mu_{z_{ik}^{\left(l\right)}}=E\left\{z_{ik}^{\left(l\right)}\right\}=\sum_{j=1,j\neq k}^{2n_{T}}{\bar{h}}_{ij}^{\left(l\right)}E\left\{{\bar{x}}_{j}^{\left(l\right)}\right\}$$

and

(6) $$\sigma_{z_{ik}^{\left(l\right)}}^{2}=\mathrm{Var}\left\{z_{ik}^{\left(l\right)}\right\}=\sum_{j=1,j\neq k}^{2n_{T}}{\left({\bar{h}}_{ij}^{\left(l\right)}\right)}^{2}\mathrm{Var}\left\{{\bar{x}}_{j}^{\left(l\right)}\right\}+\frac{\sigma^{2}}{2}.$$

The likelihood of ${\bar{x}}_{k}^{\left(l\right)}$ at each observation node $o_{i}^{\left(l\right)}$ is approximately obtained by using the Gaussian approximation of $z_{ik}^{\left(l\right)}$ as

(7) $$\mathrm{Pr}\{{\bar{y}}_{i}^{\left(l\right)}|{\bar{H}}^{\left(l\right)},{\bar{x}}_{k}^{\left(l\right)}=s\}\approx \frac{1}{\sqrt{2\pi \sigma_{z_{ik}^{\left(l\right)}}^{2}}}\exp\left(-\frac{{\left({\bar{y}}_{i}^{\left(l\right)}-{\bar{h}}_{ik}^{\left(l\right)}s-\mu_{z_{ik}^{\left(l\right)}}\right)}^{2}}{2\sigma_{z_{ik}^{\left(l\right)}}^{2}}\right),$$

where $s\in \bar{A}$. Note that $\mu_{z_{ik}^{\left(l\right)}}$ and $\sigma_{z_{ik}^{\left(l\right)}}^{2}$ are computed as

(8) $$\mu_{z_{ik}^{\left(l\right)}}=\sum_{j=1,j\neq k}^{2n_{T}}{\bar{h}}_{ij}^{\left(l\right)}\left(\sum_{s\in \bar{A}}s\cdot {\mathrm{Pr}}^{\left(i\right)}\left\{{\bar{x}}_{j}^{\left(l\right)}=s\right\}\right)$$

and

(9) $$\sigma_{z_{ik}^{\left(l\right)}}^{2}=\sum_{j=1,j\neq k}^{2n_{T}}{\left({\bar{h}}_{ij}^{\left(l\right)}\right)}^{2}\left\{\sum_{s\in \bar{A}}s^{2}\cdot {\mathrm{Pr}}^{\left(i\right)}\left\{{\bar{x}}_{j}^{\left(l\right)}=s\right\}-{\left(\sum_{s\in \bar{A}}s\cdot {\mathrm{Pr}}^{\left(i\right)}\left\{{\bar{x}}_{j}^{\left(l\right)}=s\right\}\right)}^{2}\right\}+\frac{\sigma^{2}}{2},$$

where ${\mathrm{Pr}}^{\left(i\right)}\left\{{\bar{x}}_{j}^{\left(l\right)}=s\right\}$ denotes a priori probability of ${\bar{x}}_{j}^{\left(l\right)}$ at the observation node $o_{i}^{\left(l\right)}$. The extrinsic probability of ${\bar{x}}_{k}^{\left(l\right)}$ at each observation node $o_{i}^{\left(l\right)}$ is obtained as [22]

(10) $$\mathrm{Pr}\left\{{\bar{x}}_{k}^{\left(l\right)}=s|{\bar{H}}^{\left(l\right)},{\bar{y}}_{\setminus i}^{\left(l\right)}\right\}=\kappa \prod_{j=1,j\neq i}^{2n_{R}}\mathrm{Pr}\left\{{\bar{y}}_{j}^{\left(l\right)}|{\bar{H}}^{\left(l\right)},{\bar{x}}_{k}^{\left(l\right)}=s\right\}\cdot \mathrm{Pr}\left\{{\bar{x}}_{k}^{\left(l\right)}=s\right\},$$

where κ is a constant. As simple notations, we let $\alpha_{ik}^{\left(l\right)}\left(s\right)$ and $\beta_{ki}^{\left(l\right)}\left(s\right)$ denote the likelihood and the extrinsic probability, respectively, of ${\bar{x}}_{k}^{\left(l\right)}=s$ at the observation node $o_{i}^{\left(l\right)}$, i.e., $\alpha_{ik}^{\left(l\right)}\left(s\right)=\mathrm{Pr}\left\{{\bar{y}}_{i}^{\left(l\right)}|{\bar{H}}^{\left(l\right)},{\bar{x}}_{k}^{\left(l\right)}=s\right\}$ and $\beta_{ki}^{\left(l\right)}\left(s\right)=\mathrm{Pr}\left\{{\bar{x}}_{k}^{\left(l\right)}=s|{\bar{H}}^{\left(l\right)},{\bar{y}}_{\setminus i}^{\left(l\right)}\right\}$.

In the iterative process, the extrinsic probability replaces the role of a priori probability. In other words, ${\mathrm{Pr}}^{\left(i\right)}\left\{{\bar{x}}_{j}^{\left(l\right)}=s\right\}$ in Equations (8) and (9) are replaced by $\beta_{ji}^{\left(l\right)}\left(s\right)$. Then, $\alpha_{ik}^{\left(l\right)}\left(s\right)$ is computed at the observation node $o_{i}^{\left(l\right)}$ by using $\beta_{ji}^{\left(l\right)}\left(s\right)$, j≠k, via $\mu_{z_{ik}^{\left(l\right)}}$ and $\sigma_{z_{ik}^{\left(l\right)}}^{2}$ based on Equations (7)–(9) and delivered to the middle node $m_{k}^{\left(l\right)}$. Note that $\beta_{ki}^{\left(l\right)}\left(s\right)$ is computed at the middle node $m_{k}^{\left(l\right)}$ by using $\alpha_{jk}^{\left(l\right)}\left(s\right)$, j≠i, as in Equation (10), and delivered to the observation node $o_{i}^{\left(l\right)}$. Consequently, $\alpha_{ik}^{\left(l\right)}\left(s\right)$ and $\beta_{ki}^{\left(l\right)}\left(s\right)$ are updated in a recursive manner through detection iterations.

At the end of detection iterations, the log-likelihood ratios (LLR) of coded bits are computed at middle nodes in the following manner and delivered to the decoder. We suppose that a variable node $v=v_{r}$ represents the *t*th bit in the bit-stream generating ${\bar{x}}_{k}^{\left(l\right)}$, which results in $r=\left(l-1\right)\cdot n_{T}\log_{2}M_{o}+\left(k-1\right)\cdot \log_{2}\sqrt{M_{o}}+t$. Then, the LLR of the coded bit $u_{r}$ corresponding to the variable node $v_{r}$ is defined by $L_{v_{r}}=\log\frac{\mathrm{Pr}\{u_{r}=0\}}{\mathrm{Pr}\{u_{r}=1\}}$ and obtained at the middle node $m_{k}^{\left(l\right)}$ as [22]

(11) $$L_{v_{r}}=\log\frac{\sum_{s\in S_{t}^{-}}\mathrm{Pr}\left\{{\bar{x}}_{k}^{\left(l\right)}=s|{\bar{H}}^{\left(l\right)},{\bar{y}}^{\left(l\right)}\right\}}{\sum_{s\in S_{t}^{+}}\mathrm{Pr}\left\{{\bar{x}}_{k}^{\left(l\right)}=s|{\bar{H}}^{\left(l\right)},{\bar{y}}^{\left(l\right)}\right\}}=\log\frac{\sum_{s\in S_{t}^{-}}\prod_{i=1}^{2n_{R}}\alpha_{ik}^{\left(l\right)}\left(s\right)}{\sum_{s\in S_{t}^{+}}\prod_{i=1}^{2n_{R}}\alpha_{ik}^{\left(l\right)}\left(s\right)},$$

where $S_{t}^{-}=\left\{s\right|$ is the *t*th bit of a bit-stream generating a symbol *s* is 0} and $S_{t}^{+}=\left\{s\right|$ is the *t*th bit of a bit-stream generating a symbol *s* is 1}. In the last equality of Equation (11), we use $\mathrm{Pr}\left\{{\bar{x}}_{k}^{\left(l\right)}=s|{\bar{H}}^{\left(l\right)},{\bar{y}}^{\left(l\right)}\right\}\propto \prod_{i=1}^{2n_{R}}\mathrm{Pr}\left\{{\bar{y}}_{i}^{\left(l\right)}|{\bar{H}}^{\left(l\right)},{\bar{x}}_{k}^{\left(l\right)}=s\right\}$. The messages $L_{v_{r}}$ obtained at middle nodes are delivered to the decoder to be used in the sum-product decoding.

Next, consider the operation of sum-product decoding. Let $L_{v_{r}c}$ and $L_{cv_{r}}$ denote the message flowing from the variable node $v_{r}$ to the check node *c* and the message flowing from the check node *c* to the variable node $v_{r}$, respectively. These messages are updated in an iterative manner by [29,30]

(12) $$L_{v_{r}c}=L_{v_{r}}+\sum_{c^{\prime }\in C_{v_{r}}\setminus c}L_{c^{\prime }v_{r}}$$

and

(13) $$L_{cv_{r}}=\prod_{v^{\prime }\in V_{c}\setminus v_{r}}\mathrm{sign}\left(L_{v^{\prime }c}\right)\cdot \phi \left(\sum_{v^{\prime }\in V_{c}\setminus v_{r}}\phi \left(|L_{v^{\prime }c}|\right)\right),$$

where $\phi \left(x\right)=\log\left(\frac{\exp(x)+1}{\exp(x)-1}\right)$. Note that $C_{v_{r}}\setminus c$ denotes the set of check nodes except *c* connected to the variable node $v_{r}$ and $V_{c}\setminus v_{r}$ denotes the set of variable nodes except $v_{r}$ connected to the check node *c*. At the end of decoding iterations, the LLR message of the *t*th bit in the bit-stream generating ${\bar{x}}_{k}^{\left(l\right)}$ is computed as $L_{k}^{\left(l\right)}\left(t\right)=\sum_{c\in C_{v_{r}}}L_{cv_{r}}$ and delivered to the middle node $m_{k}^{\left(l\right)}$ in the detector. At the beginning of the next detection iteration, the probability $\mathrm{Pr}\{{\bar{x}}_{k}^{\left(l\right)}=s\}$ is computed at the middle node $m_{k}^{\left(l\right)}$ by

(14) $$\mathrm{Pr}\left\{{\bar{x}}_{k}^{\left(l\right)}=s\right\}=\prod_{t=1}^{\log_{2}\sqrt{M_{o}}}\frac{\exp\left(\left(1-s\left(t\right)\right)\cdot L_{k}^{\left(l\right)}\left(t\right)\right)}{1+\exp\left(L_{k}^{\left(l\right)}\left(t\right)\right)},$$

and used in the detector as in Equation (10), where s(t) denotes the value of the *t*th bit in the bit-stream generating a symbol *s*.

**Figure 2:**
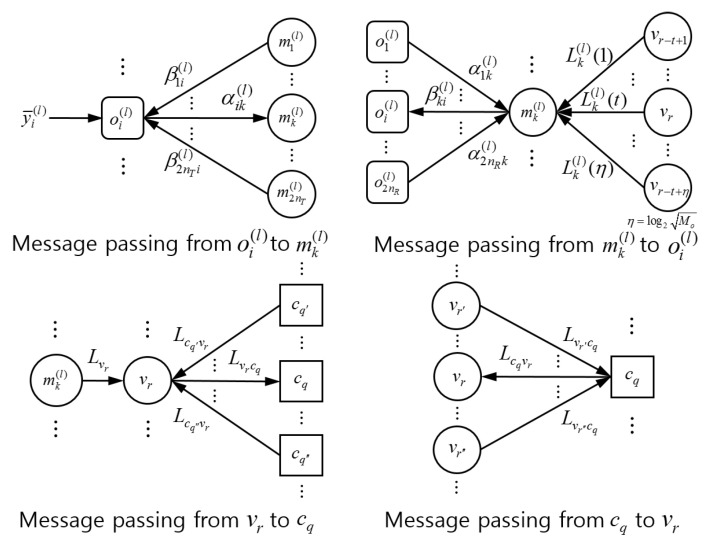
Message passing in the JDD of receiver for LDPC coded massive MIMO system.

After $N_{g}$ global iterations, the decision on bits is made such that the coded bit $u_{r}$ is estimated as 1 if $L_{v_{r}}+L_{k}^{\left(l\right)}\left(t\right)<0$ and as 0 otherwise, where $r=\left(l-1\right)\cdot n_{T}\log_{2}M_{o}+\left(k-1\right)\cdot \log_{2}\sqrt{M_{o}}+t$. The overall procedure of JDD is presented in Algorithm 1 and the FG-GAI BP detection is summarized in Algorithm 2. Message flows between component nodes of the JDD are illustrated in Figure 2.

**Algorithm 1:** Joint Detection and Decoding (JDD).

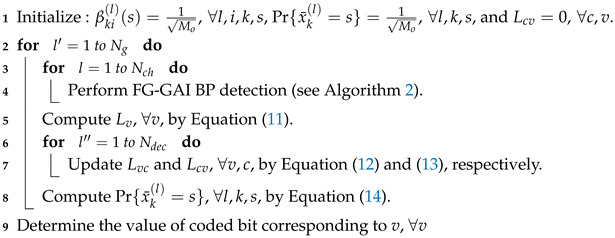



**Algorithm 2:** FG-GAI BP detection.

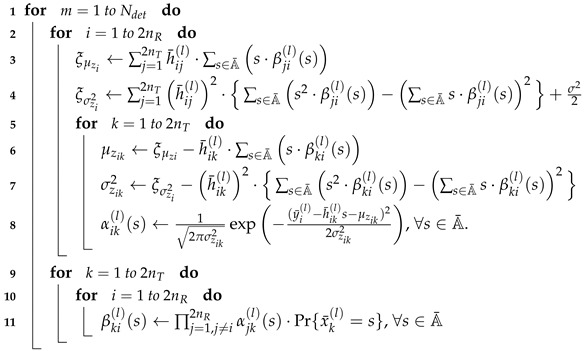



Let us think about the computational complexity of FG-GAI BP detection in terms of the number of multiplications. We focus on one detection iteration for one channel use with a given modulation. It is easily inferred from Algorithm 2 that the FG-GAI BP detection requires a computational complexity of $O\left(n_{T}n_{R}\right)$. For a brief comparison, we consider some other SISO MIMO detectors such as BP detector [21], SISO MMSE detector, tree-searching detector such as sphere decoding (SD) aided max-log method [26] and subspace marginalization with interference suppression (SUMIS) detector [26]. These detectors require computational complexities of $O\left(n_{T}^{2}n_{R}\right)$. It is clear that the FG-GAI-BP detector requires lower computational complexity than other SISO MIMO detectors under comparison.

### 3.2. Analysis of Joint Detection and Decoding

**Figure 3:**
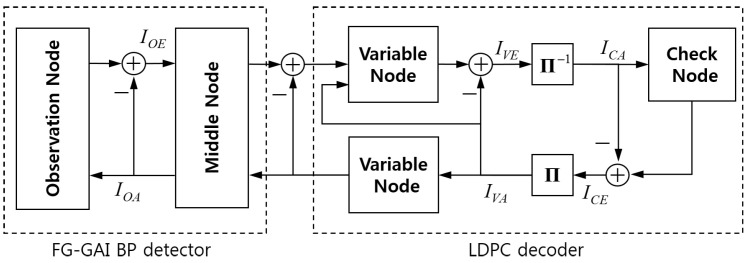
The JDD process in terms of EXIT characteristics.

We analyze the behavior of JDD in the receiver of coded massive MIMO system in terms of mutual information transfer characteristics, so-called EXIT characteristics, in component units. We focus on the mutual information between coded bits generating transmit symbols and corresponding message variables. In fact, $\alpha_{ik}^{\left(l\right)}\left(s\right)$ and $\beta_{ki}^{\left(l\right)}\left(s\right)$ at the observation node $o_{i}^{\left(l\right)}$ contain information regarding the transmit symbol ${\bar{x}}_{k}^{\left(l\right)}$. Thus, for the bit-level EXIT analysis mentioned above, we define new LLR messages of coded bits at observation nodes. Let us consider the *t*th bit of a coded bit-stream mapped to ${\bar{x}}_{k}^{\left(l\right)}$ and define two LLR messages of this bit at the observation node $o_{i}^{\left(l\right)}$. The first LLR is $L_{ki}^{\left(l\right)}\left(t\right)=\log\frac{\sum_{s\in S_{t}^{-}}\beta_{ki}^{\left(l\right)}\left(s\right)}{\sum_{s\in S_{t}^{+}}\beta_{ki}^{\left(l\right)}\left(s\right)}$ sent from $m_{k}^{\left(l\right)}$ to $o_{i}^{\left(l\right)}$, and the second LLR is $L_{ik}^{\left(l\right)}\left(t\right)=\log\frac{\sum_{s\in S_{t}^{-}}\alpha_{ik}^{\left(l\right)}\left(s\right)}{\sum_{s\in S_{t}^{+}}\alpha_{ik}^{\left(l\right)}\left(s\right)}$ sent from $o_{i}^{\left(l\right)}$ to $m_{k}^{\left(l\right)}$, where $k=1,\cdots ,2n_{T}$. We let $L_{o}^{in}$ and $L_{o}^{out}$ denote random variables representing $L_{ki}^{\left(l\right)}\left(t\right)$ and $L_{ik}^{\left(l\right)}\left(t\right)$, respectively, for all k,i,t, and let *U* denote the corresponding coded bit. We suppose all LLR messages are independent and normally distributed. For each observation node, we define $I_{OA}=I\left(U;L_{o}^{in}\right)$ and $I_{OE}=I\left(U;L_{o}^{out}\right)$. We define $I_{VA}=I\left(U;L_{cv}\right)$ and $I_{VE}=I\left(U;L_{vc}\right)$ at variable nodes, where $L_{cv}$ and $L_{vc}$ are incoming and outgoing messages at variable nodes, respectively. We also define $I_{CA}=I\left(U;L_{vc}\right)$ and $I_{CE}=I\left(U;L_{cv}\right)$ at check nodes, where $L_{vc}$ and $L_{cv}$ are incoming and outgoing messages at check nodes, respectively. Allowing slight abuse of notation, we use $I_{VA}\left(d_{c}\right)$ and $I_{CE}\left(d_{c}\right)$ to denote the mutual information between *U* and $L_{cv}$ delivered from degree-$d_{c}$ check nodes to a variable node. We also use $I_{VE}\left(d_{v}\right)$ and $I_{CA}\left(d_{v}\right)$ to denote the mutual information between *U* and $L_{vc}$ delivered from degree-$d_{v}$ variable nodes to a check node. We depict the resultant iterative JDD process represented by transfer blocks of mutual information as in Figure 3.

Consider a degree-$d_{v}$ variable node that is connected to $d_{v}$ check nodes and $2n_{R}$ observation nodes via a corresponding middle node. The variable node sums up all incoming messages except one from a target node and sends the result to the target node. Thus, $L_{o}^{in}$ is obtained by summing up $2n_{R}-1$ copies of $L_{o}^{out}$ and $d_{v}$ copies of $L_{cv}$. It follows that the variance of $L_{o}^{in}$ is obtained by adding the variance of $L_{o}^{out}$ multiplied by $2n_{R}-1$ and the variance of $L_{cv}$ multiplied by $d_{v}$. By defining $J(\sigma_{X})$ as [33]

(15) $$J\left(\sigma_{X}\right)=1-\int_{-\infty }^{\infty }\frac{e^{-{\left(\xi -\sigma_{X}^{2}/2\right)}^{2}/2\sigma_{X}^{2}}}{\sqrt{2\pi \sigma_{X}^{2}}}\cdot \log_{2}\left[1+e^{-\xi }\right]d\xi ,$$

we obtain $I\left(U;X\right)=J\left(\sigma_{X}\right)$, where $\sigma_{X}^{2}$ is the variance of a normally distributed random variable *X*. Then, $I_{OA}$ is obtained as a function of $d_{v}$ as

(16) $$I_{OA}\left(d_{v}\right)\;=\;J\left(\;\sqrt{\left(2n_{R}-1\right)\;\cdot \;{\left[J^{-1}\left(I_{OE}\left(d_{v}\right)\right)\right]}^{2}+d_{v}\cdot {\left[J^{-1}\left({\bar{I}}_{VA}\right)\right]}^{2}}\right),$$

where ${\bar{I}}_{VA}=\sum_{d_{c}=2}^{d_{c,\max}}\rho_{d_{c}}\cdot I_{VA}\left(d_{c}\right)$ is the average of $I_{VA}\left(d_{c}\right)$ over $d_{c}$, $\rho_{d_{c}}$ denotes the fraction of edges that are connected to check nodes of degree $d_{c}$, and $d_{c,\max}$ denotes the maximum degree of check node. Let us define the EXIT function between $I_{OA}$ and $I_{OE}$ as

(17) $$I_{OE}\left(d_{v}\right)=f_{O}\left(I_{OA}\left(d_{v}\right),\frac{E_{b}}{N_{0}}\right),$$

where $I_{OE}$ is also a function of $d_{v}$ due to the dependency of $I_{OA}$ on $d_{v}$. Note that $f_{O}\left(\cdot \right)$ is obtained by Monte Carlo simulation [33]. The LLR message $L_{vc}$ sent from the variable node to check nodes is obtained by summing up $2n_{R}$ copies of $L_{o}^{out}$ and $d_{v}-1$ copies of $L_{cv}$. Then, the variance of $L_{vc}$ is obtained by adding the variance of $L_{o}^{out}$ multiplied by $2n_{R}$ and the variance of $L_{cv}$ multiplied by $d_{v}-1$. It follows that

(18) $$I_{VE}\left(d_{v}\right)\;=\;J\left(\;\sqrt{2n_{R}\cdot {\left[J^{-1}\left(I_{OE}\left(d_{v}\right)\right)\right]}^{2}+\left(d_{v}-1\right)\;\cdot \;{\left[J^{-1}\left({\bar{I}}_{VA}\right)\right]}^{2}}\right).$$

In the case of irregular distribution of $d_{v}$, we define averages of $I_{VE}\left(d_{v}\right)$ and $I_{OE}\left(d_{v}\right)$ over $d_{v}$ as

(19) $${\bar{I}}_{VE}=\sum_{d_{v}=2}^{d_{v,\max}}\lambda_{d_{v}}\cdot I_{VE}\left(d_{v}\right)$$

and

(20) $${\bar{I}}_{OE}=\sum_{d_{v}=2}^{d_{v,\max}}\lambda_{d_{v}}\cdot I_{OE}\left(d_{v}\right),$$

respectively, where $\lambda_{d_{v}}$ denotes the fraction of edges that are connected to variable nodes of degree $d_{v}$ and $d_{v,\max}$ denotes the maximum degree of variable node.

Let us consider a degree-$d_{c}$ check node and define the EXIT function from ${\bar{I}}_{CA}$ to $I_{CE}$ as [33]

(21) $$I_{CE}\left(d_{c}\right)\approx 1-J\left(\sqrt{d_{c}-1}\cdot J^{-1}\left(1-{\bar{I}}_{CA}\right)\right),$$

where ${\bar{I}}_{CA}=\sum_{d_{v}=1}^{d_{v,max}}\lambda_{d_{v}}\cdot I_{CA}\left(d_{v}\right)$ is the average of $I_{CA}\left(d_{v}\right)$ over $d_{v}$. In the case of irregular distribution of $d_{c}$, we define the average of $I_{CE}\left(d_{c}\right)$ over $d_{c}$ as

(22) $${\bar{I}}_{CE}=\sum_{d_{c}=2}^{d_{c,\max}}\rho_{d_{c}}\cdot I_{CE}\left(d_{c}\right).$$

The density evolution of messages flowing in the JDD process in terms of EXIT characteristics is summarized in Algorithm 3.

**Figure 4:**
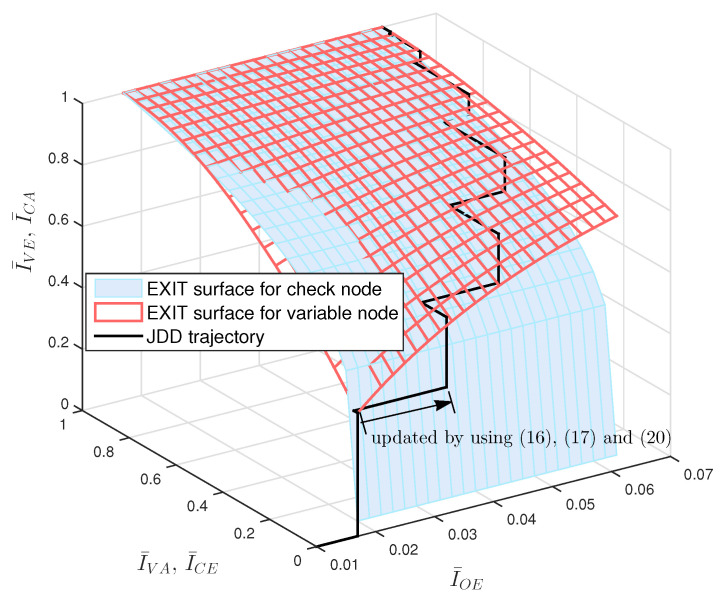
The 3-D EXIT chart and trajectory of JDD process for (3, 6)-regular LDPC coded massive MIMO system with 4-QAM over 16×16 MIMO channel at $E_{b}/N_{0}=5$ [dB], where ${\bar{I}}_{OE}$ denotes the mutual information per observation node.

We can obtain the 3-D EXIT chart of JDD process by using Equations (16)–(22). The EXIT surface for variable nodes is obtained by using Equations (18)–(20). The EXIT surface for check nodes is obtained by stretching along the ${\bar{I}}_{OE}$-axis the 2-D EXIT function from ${\bar{I}}_{CA}$ to ${\bar{I}}_{CE}$ obtained by Equations (21) and (22). As an example, we plot in Figure 4 the 3-D EXIT chart of JDD process for (3, 6)-regular LDPC coded massive MIMO systems with $N_{det}=1$ and $N_{dec}=1$, where coded bits are 4-QAM modulated and transmitted over 16×16 MIMO channel. We also plot in Figure 4 the JDD trajectory obtained by Algorithm 3, where the update for ${\bar{I}}_{OE}$ is computed by Equations (16), (17) and (20). It is observed that the JDD trajectory is formed between two EXIT surfaces. If the JDD trajectory approaches a point with ${\bar{I}}_{VE}=1$ at a certain $E_{b}/N_{0}$, this implies that the JDD converges and the decoding succeeds at this $E_{b}/N_{0}$. The minimum value of $E_{b}/N_{0}$ resulting in the JDD trajectory approaching ${\bar{I}}_{VE}=1$ is called the threshold. We can find the threshold value of LDPC coded massive MIMO system by using Algorithm 3 and visualize the JDD behavior by using the 3-D EXIT chart.

**Algorithm 3:** Density evolution in terms of EXIT characteristics.

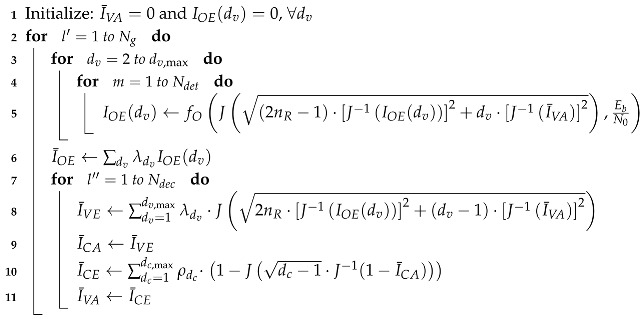



## 4. Design of LDPC Coded Massive MIMO System

In general, LDPC codes are designed through two steps: the optimization of degree distributions and the placement of edges between variable nodes and check nodes. Degree distributions of variable nodes and check nodes from the edge perspective are represented in the form of polynomials as [29]

(23) $$\lambda \left(x\right)=\sum_{d_{v}=2}^{d_{v,\max}}\lambda_{d_{v}}x^{d_{v}-1}\;\mathrm{and}\;\rho \left(x\right)=\sum_{d_{c}=2}^{d_{c,\max}}\rho_{d_{c}}x^{d_{c}-1},$$

respectively. Then, the code rate ***R*** is given by [29]

(24) $$R\left(\lambda ,\rho \right)=1-\frac{\sum_{d_{c}=2}^{d_{c,\max}}\rho_{d_{c}}/d_{c}}{\sum_{d_{v}=2}^{d_{v,\max}}\lambda_{d_{v}}/d_{v}},$$

where $\lambda =\{\lambda_{2},\cdots ,\lambda_{d_{v,max}}\}$ and $\rho =\{\rho_{2},\cdots ,\rho_{d_{c,max}}\}$. In the first step of designing LDPC codes, we first determine degree distributions to maximize the code rate guaranteeing the convergence of JDD at a given $E_{b}/N_{0}$ by using the density evolution algorithm. By repeating the same procedure for various values of $E_{b}/N_{0}$, we find the smallest $E_{b}/N_{0}$ resulting in the maximum code rate exceeding the target code rate. Such $E_{b}/N_{0}$ is called the threshold, and the corresponding degree distributions are considered optimal. In the second step of LDPC code design, we place edges between variable nodes and check nodes based on given optimal degree distributions to satisfy the following criteria [29]:

(a) Avoid short cycles involving only degree-2 variable nodes.
(b) Length-4 cycles need to be avoided.

These criteria can be satisfied by using the progressive edge growth (PEG) algorithm [40]. When we construct finite-length LDPC codes, the following criterion needs to be satisfied additionally:

(c) All degree-2 variable nodes need to represent only non-systematic bits.

Let $\Lambda_{2}$ denote the number of degree-2 variable nodes. Then, $\Lambda_{2}\leq N-K$, or equivalently,

(25) $$\lambda_{2}\leq 2\sum_{d_{c}=2}^{d_{c,\max}}\rho_{d_{c}}/d_{c}$$

is a necessary condition to satisfy Criterion (c). Thus, we need to take into consideration the condition in Equation (25) when determining degree distributions in the first step of LDPC code design.

**Figure 5:**
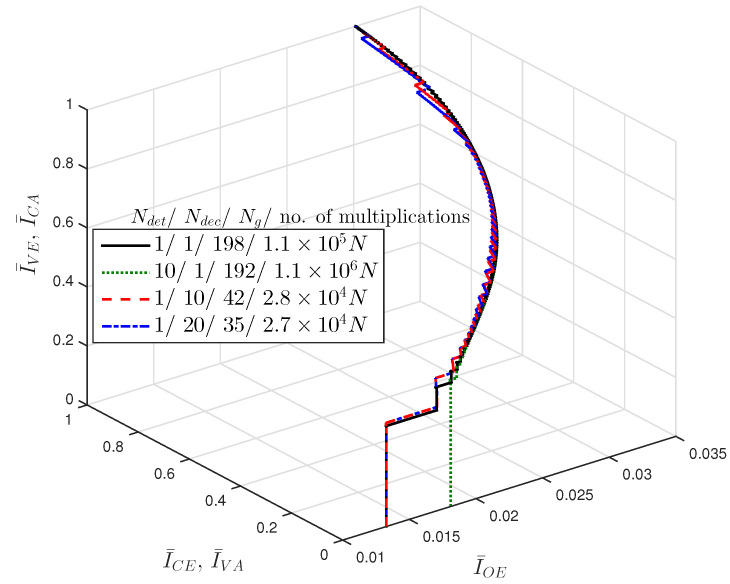
JDD trajectories of LDPC coded massive MIMO system with R=1/2, $n_{T}=n_{R}=16$ and $E_{b}/N_{0}=1.50$ [dB].

Since the computational complexity is a major concern, we need to design the coded massive MIMO system such that the error correction capability is maximized with a given amount of computational complexity. As introduced above, one global iteration of JDD consists of $N_{det}$ detection iterations and $N_{dec}$ decoding iterations. We can speed up the convergence of JDD by controlling the ratio of $N_{det}$ and $N_{dec}$ in one global iteration. In Table 1, we list the approximate numbers of multiplications and additions required to compute various messages in the JDD process, where we assume that exp(·), log(·) and ϕ(·) are obtained in a look-up-table manner. Total approximate numbers of operations required by $N_{g}$ global iterations of JDD are listed in Table 2, which are also approximated as functions of *N* if *N* is large enough. We obtain JDD trajectories of LDPC coded massive MIMO system by using Algorithm 3 for different combinations of $N_{det}$ and $N_{dec}$, and plot the results in Figure 5, where $M_{o}=4$ and $n_{T}=n_{R}=16$. For each trajectory, we specify the approximate number of required multiplications. It is observed that JDD trajectories may converge to the same values of ${\bar{I}}_{VE}$ and ${\bar{I}}_{CE}$, resulting in the same BER performance, with different computational complexities depending on the combination of $N_{det}$ and $N_{dec}$. This verifies the importance of the JDD strategy represented by $N_{det}$ and $N_{dec}$ to operate the JDD efficiently.

Let us focus on the number of multiplications for a simple analysis of computational complexity. It is clear from Table 2 that the number of multiplications required for detection is much higher than that for decoding, especially with large $n_{T}$ and $n_{R}$. Thus, increasing $N_{det}$ results in much higher computational complexity than increasing $N_{dec}$. It follows that increasing $N_{det}$ results in the significant decrease of $N_{g}$ to maintain the total amount of computational complexity. With a small $N_{g}$, the JDD does not converge sufficiently so that a low value of threshold is not attained. As a result, we fix $N_{det}=1$ and adjust $N_{dec}$ to design the JDD strategy, where $N_{g}$ is determined by $N_{dec}$ with other parameters given. We call the JDD with $N_{det}=N_{dec}=1$ the global iteration only (GIO) JDD.

Consider a LDPC coded massive MIMO system using GIO JDD with $N_{g}=N_{g}^{GIO}$ as a reference system. The degree distributions of LDPC codes and the JDD strategy in a proposed coded massive MIMO system are determined in the following manner. First, we choose candidate values of $N_{dec}$ resulting in the number of multiplications equivalent to that of GIO JDD. For each $E_{b}/N_{0}$, we perform the following optimization for all candidate values of $N_{dec}$:

(26) $$\begin{array}{l} \max_{\lambda ,\rho }\;\;R(\lambda ,\rho )\\ \;s.t.\;\;{\bar{I}}_{VE}=1\;\;\mathrm{after}\;\mathrm{running}\;\mathrm{Algorithm}\;3,\\ \;\lambda_{2}\leq 2\sum_{d_{c}=2}^{d_{c,\max}}\rho_{d_{c}}/d_{c},\\ \;\sum_{d_{c}=2}^{d_{c,\max}}\rho_{d_{c}}=\sum_{d_{v}=2}^{d_{v,\max}}\lambda_{d_{v}}=1\;\mathrm{with}\;\rho_{d_{c}},\lambda_{d_{v}}\geq 0, \end{array}$$

where the first constraint guarantees the convergence of JDD and the second constraint is used to satisfy Criterion (c) introduced above. The lowest $E_{b}/N_{0}$, at which there exist $N_{dec}$ such that the maximum R(λ,ρ) exceeds the target rate, is called the threshold and denoted by ${\left(E_{b}/N_{0}\right)}^{*}$. Degree distributions and $N_{dec}$ resulting in the lowest threshold are determined as optimal parameters of LDPC codes and the JDD strategy, respectively. Then, we construct the parity check matrix of LDPC codes based on optimally determined degree distributions by using the PEG algorithm.

## 5. Numerical Results

We considered LDPC coded massive MIMO systems over 16×16, 64×64 and 256×256 channels with code rates of R=0.5 and 0.75. Coded bits were mapped to 4-QAM transmit symbols by Gray-mapping. We considered a coded massive MIMO system using GIO JDD with an arbitrary $N_{g}^{GIO}$ as a reference system, based on which the number of multiplications was evaluated as a function of *N* from Table 2. Then, we chose candidate values of $N_{dec}$ of the proposed JDD strategy such that the total number of multiplications with $N_{det}=1$ was equivalent to that of GIO JDD, where $N_{g}$ was determined by $N_{dec}$ with other given parameters. We solved the optimization problem in Equation (26) for each $E_{b}/N_{0}$ and $N_{dec}$ by using the differential evolution algorithm [41]. We found degree distributions and the value of $N_{dec}$ resulting in the smallest threshold and the rate exceeding the target rate. Note that, in determining degree distributions of LDPC codes, we used the concentrated check node degree distribution [30], i.e., $\rho \left(x\right)=\rho_{d_{c}}x^{d_{c}}+\left(1-\rho_{d_{c}}\right)x^{d_{c}+1}$. We constructed the parity check matrix of LDPC codes by using the degree distribution and the PEG algorithm. Then, we generated LDPC codes from the obtained parity check matrix and performed BER simulations.

### 5.1. Convergence Speed of JDD Strategy

**Figure 6:**
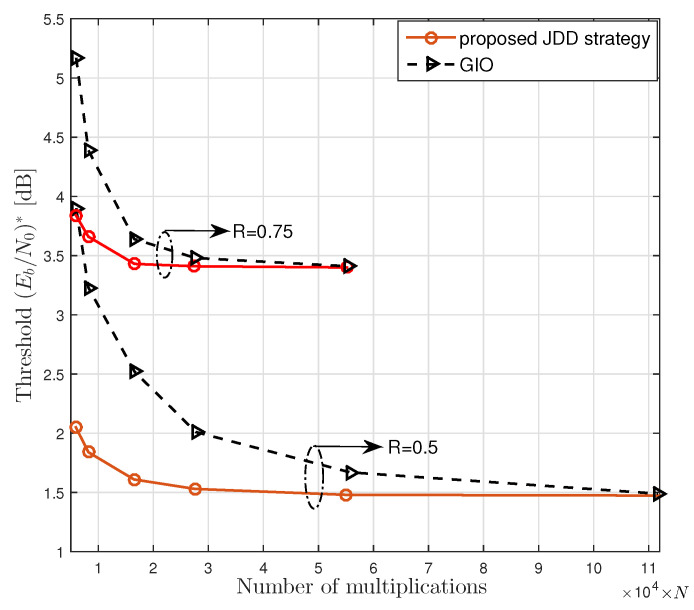
The threshold of the LDPC coded massive MIMO system with the proposed JDD strategy and with the GIO JDD obtained by the density evolution algorithm given in Algorithm 3, where R=0.5 and R=0.75 were considered with $n_{T}=n_{R}=16$.

We show the benefit of using the proposed JDD strategy in view of the threshold with respect to the amount of computational complexity. In Figure 6, we plot the threshold ${\left(E_{b}/N_{0}\right)}^{*}$ obtained by the density evolution algorithm given in Algorithm 3 for the proposed JDD strategy and GIO JDD over 16×16 channel with various numbers of multiplications. It was observed that the proposed JDD strategy converged to the lowest threshold faster than GIO JDD. It was also observed that the proposed JDD strategy resulted in a lower threshold than GIO JDD for a given amount of computational complexity.

### 5.2. Performance Comparison without Complexity Constraint

We next considered the JDD without constraint on the amount of computational complexity. We investigated the threshold and BER obtained with different values of $N_{dec}$, where the case of $N_{dec}=1$ corresponded to the reference system using GIO JDD. In Table 3, we list optimal degree distributions of LDPC codes and resultant thresholds for some values of *R*, $N_{dec}$ and $d_{v,\max}$ with $n_{T}=n_{R}=16$. It was observed that the proposed JDD strategy and the GIO JDD resulted in the same threshold for given $d_{v,\max}$ and *R*.

**Figure 7:**
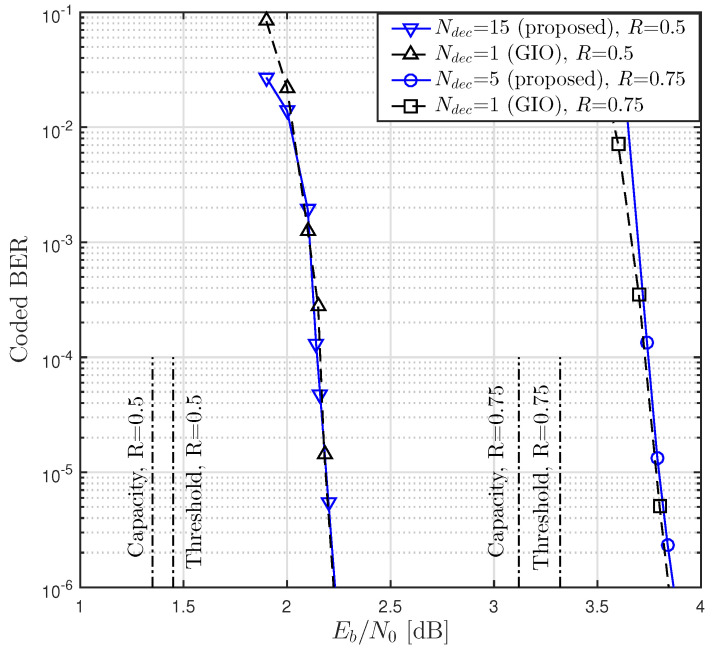
BER performances of LDPC coded massive MIMO systems over 16×16 channel with various $N_{dec}$ and sufficiently high amount of computational complexity, where *N* = 64,000.

In Figure 7, we plot BER performances of coded massive MIMO systems with *N* = 64,000 over 16×16 channel for some values of $N_{dec}$ and sufficiently large $N_{g}$. The degree distributions listed in Table 3 corresponding to $d_{v,\max}=24$ and $d_{v,\max}=20$ for R=0.5 and R=0.75, respectively, were used for generating LDPC codes. We used $N_{g}=200$ and $N_{g}=85$ for $N_{dec}=1$ and $N_{dec}=15$, respectively, when R=0.5 and we used $N_{g}=100$ and $N_{g}=90$ for $N_{dec}=1$ and $N_{dec}=5$, respectively, when R=0.75. It was observed that the proposed JDD strategy and GIO JDD showed similar BER performances if a sufficiently high amount of computational complexity was allowed. This result agreed with the threshold analysis presented in Figure 6 and Table 3.

### 5.3. Performance Comparison with Complexity Constraint

**Figure 8:**
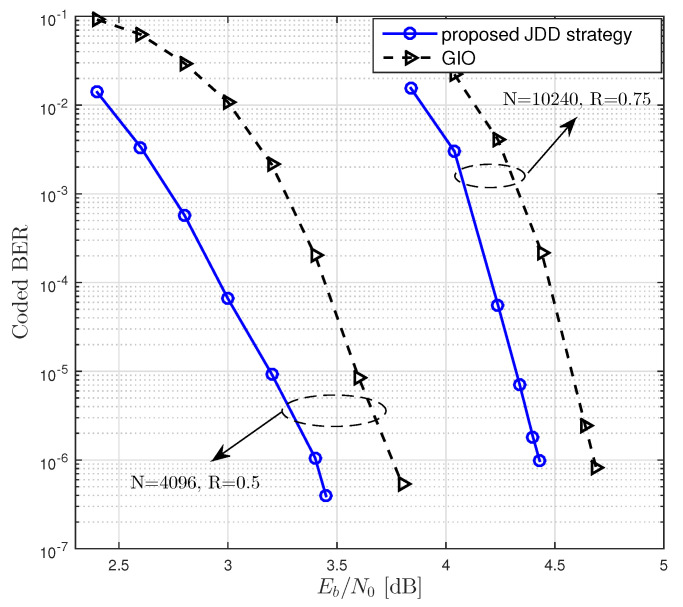
BER performances of LDPC coded massive MIMO systems equipped with the proposed JDD strategy and with GIO JDD over 16×16 channel, where N=4096 and *N* = 10,240 with R=0.5 and R=0.75, respectively, were considered.

We then considered the JDD with the finite amount of computational complexity. In Table 4 and Table 5, we list optimal degree distributions of LDPC codes in the reference system (GIO JDD) and the proposed system. It was observed that the proposed JDD strategy resulted in a lower threshold than GIO JDD. We performed BER simulations of rate-0.5 LDPC codes with N=4096 and rate-0.75 LDPC codes with *N* = 10,240, which were constructed from the degree distributions presented in Table 4 and Table 5, respectively. When R=0.5, we used degree distributions and $N_{dec}$ corresponding to $d_{v,\max}=24$ for both GIO JDD and the proposed JDD strategy. When R=0.75, we used degree distributions and $N_{dec}$ corresponding to $d_{v,\max}=3$ and $d_{v,\max}=20$ for GIO JDD and the proposed JDD strategy, respectively. In Figure 8, we plot BER performances of the LDPC coded massive MIMO system equipped with the proposed JDD strategy and with GIO JDD over 16×16 channel. It was observed that using the proposed JDD strategy resulted in a lower BER than using GIO JDD. This result agreed with the threshold analysis presented in Table 4 and Table 5. Consequently, the jointly designed LDPC codes and JDD strategy could result in the lower BER of coded massive MIMO system by using the equivalent amount of computational complexity.

**Figure 9:**
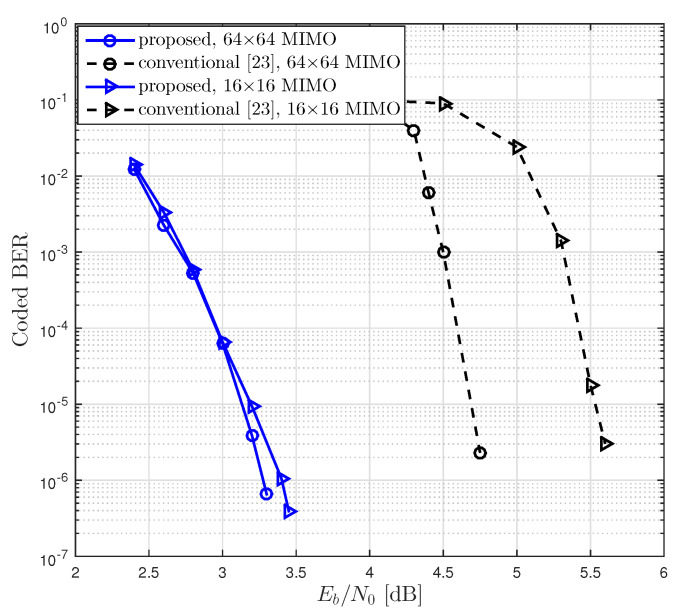
BER performances of coded massive MIMO systems with the proposed LDPC codes and JDD strategy and with the conventional LDPC codes and GIO JDD, where R=0.5 and *N* = 4096 over 16×16 and 64×64 channels were considered.

**Figure 10:**
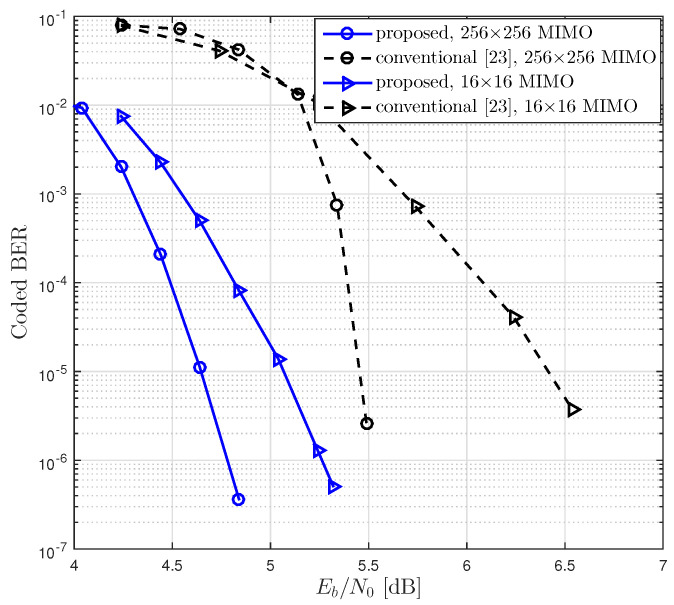
BER performances of coded massive MIMO systems with the proposed LDPC codes and JDD strategy and with the conventional LDPC codes and GIO JDD, where R=0.75 and N=2048 over 16×16 and 256×256 channels were considered.

As shown in Figure 9 and Figure 10, we compared BER performances of the proposed LDPC coded massive MIMO system and the conventional system with various numbers of antennas, where the conventional system was equipped with the conventional LDPC codes and GIO JDD [38]. As the proposed system, we constructed the rate-0.5 LDPC codes with N=4096 using the degree distribution corresponding to $d_{v,\max}=24$ in Table 4 and the rate-0.75 LDPC codes with N=2048 using the degree distribution corresponding to $d_{v,\max}=3$ in Table 5. It was observed (Figure 9 and Figure 10) that the coding gain of the proposed system over the conventional one at the BER of $10^{-5}$ over 16×16 channel was about 2.3 dB and 1.4 dB for R=0.5 and R=0.75, respectively.

## 6. Conclusions

In this study, we designed the LDPC coded massive MIMO system equipped with an iterative JDD algorithm using the low-complexity FG-GAI BP detection and the sum-product decoding. We defined a factor graph representation of the LDPC coded massive MIMO system and defined updating rules for messages flowing in the JDD process. We proposed a 3-D EXIT analysis as an engineering tool for investigating the behavior of iterative JDD algorithm of coded massive MIMO receiver. Based on the EXIT analysis, we designed jointly irregular LDPC codes through the optimization of degree distributions and the JDD strategy to achieve the lowest BER with a given amount of computational complexity. The proposed 3-D EXIT analysis enables the efficient design of LDPC codes and JDD strategy for coded massive MIMO system in a joint manner. We observed that the JDD strategy and corresponding LDPC codes designed appropriately by using the proposed EXIT analysis shows a faster convergence rate than a conventional JDD algorithm. Thus, the proposed scheme results in the improved BER performance over the conventional one with the equivalent amount of computational complexity. This result is meaningful especially when the computational complexity of coded massive MIMO receiver is constrained to finite amount, which is a practical situation.

In addition to the results presented in this paper, we plan to work on the following issues as future works.

- We will perform 3-D EXIT analysis for coded massive MIMO system equipped with various kinds of MIMO detectors. Based on this, we plan to optimize the JDD strategy for each SISO MIMO detector under consideration and correspondingly design LDPC codes. Then, we will compare BER performances of coded massive MIMO systems using different MIMO detectors.
- We will study and analyze the influence of imperfect channel estimation on the performance of LDPC coded massive MIMO system. It is hard to obtain the perfect channel estimation in practice, so we need to investigate this issue thoroughly to utilize the proposed scheme in practical communication systems.
- We will work on the finite-length analysis of LDPC coded massive MIMO system. It is well known that using channel codes with short to medium blocklength results in a gentle waterfall in the BER curve [42,43,44,45]. Since practical communication systems use finite-length channel codes, we need to study this issue as a future work for the practical application of the coded massive MIMO technology.

## Figures and Tables

**Table 1:** Computational complexities for computing messages in the detector and the decoder, where ${\bar{d}}_{v}$ and ${\bar{d}}_{c}$ denote the average degree of variable nodes and check nodes, respectively.

Messages	Approximate Number of Operations	
	Addition	Multiplication
$\alpha_{ik}\left(s\right)$	$4n_{T}n_{R}\left(2\sqrt{M_{o}}+1\right)≜\Theta_{\alpha }^{A}$	$2n_{T}n_{R}\left(11\sqrt{M_{o}}+4\right)≜\Theta_{\alpha }^{M}$
$\beta_{ki}\left(s\right)$	0	$8n_{T}n_{R}\sqrt{M_{o}}≜\Theta_{\beta }^{M}$
$L_{v}$	$\left(\sqrt{M_{o}}-2\right)N≜\Theta_{v}^{A}$	$4n_{T}n_{R}\sqrt{M_{o}}+N≜\Theta_{v}^{M}$
$\mathrm{Pr}\{{\bar{x}}_{l}=s\}$	$n_{T}\sqrt{M_{o}}\log_{2}M_{o}+\bar{v}N≜\Theta_{s}^{A}$	$2n_{T}\sqrt{M_{o}}\left(\log_{2}M_{o}-1\right)≜\Theta_{s}^{M}$
$L_{vc}$	$\left(2{\bar{d}}_{v}+1\right)N≜\Theta_{vc}^{A}$	0
$L_{cv}$	$2{\bar{d}}_{c}\left(N-K\right)≜\Theta_{cv}^{A}$	$3{\bar{d}}_{c}\left(N-K\right)≜\Theta_{cv}^{M}$

**Table 2:** Total approximate number of operations required for overall JDD.

Operation	Total Approximate Number of Operations
Addition	$N_{g}\left[N_{ch}N_{det}\Theta_{\alpha }^{A}+\Theta_{v}^{A}+N_{dec}\left(\Theta_{vc}^{A}+\Theta_{cv}^{A}\right)\right]+\left(N_{g}-1\right)N_{ch}\Theta_{s}^{A}$
	$\approx {\bar{d}}_{v}/\left(n_{T}\log_{2}M_{o}\right)N_{g}N^{2}+\left\{\left(8n_{R}\sqrt{M_{o}}/\log_{2}M_{o}\right)N_{det}+2\left({\bar{d}}_{v}+{\bar{d}}_{c}\right)N_{dec}\right\}N_{g}N$
Multiplication	$N_{g}\left[N_{ch}\left\{N_{det}\Theta_{\alpha }^{M}+\left(N_{det}-1\right)\Theta_{\beta }^{M}\right\}+\Theta_{v}^{M}+N_{dec}\Theta_{cv}^{M}\right]+\left(N_{g}-1\right)N_{ch}\left(\Theta_{s}^{M}+\Theta_{\beta }^{M}\right)$
	$\approx \left(30n_{R}\sqrt{M_{o}}N_{det}+\left(3{\bar{d}}_{c}\left(1-R\right)\log_{2}M_{o}\right)N_{dec}\right)N_{g}N$

**Table 3:** Optimal degree distributions of LDPC codes in a coded massive MIMO system over 16×16 channel for some values of $N_{dec}$, $d_{v,\max}$ and *R*. We also specified the threshold ${\left(\frac{E_{b}}{N_{0}}\right)}_{dB}^{*}$, the capacity $\Gamma_{dB}^{*}$ and their gap, where the capacity of MIMO channel with $M_{o}$-ary input was obtained by using a formula given in [34].

R	0.5				0.75			
$d_{v,\max}$	12	24	12	24	12	20	12	20
$\lambda_{2}$	0.31177	0.26138	0.31162	0.26137	0.17195	0.14485	0.17195	0.14485
$\lambda_{3}$	0.39520	0.30763	0.39486	0.30766	0.76044	0.62119	0.76044	0.62119
$\lambda_{8}$		0.12685		0.12684				
$\lambda_{12}$	0.29304		0.29352		0.06761		0.06761	
$\lambda_{20}$						0.23396		0.23396
$\lambda_{24}$		0.30414		0.30413				
$\rho_{6}$	0.54707		0.54404					
$\rho_{7}$	0.45293	0.31868	0.45596	0.31868				
$\rho_{8}$		0.68132		0.68132				
$\rho_{11}$					0.34890		0.34889	
$\rho_{12}$					0.65110		0.65111	
$\rho_{13}$						0.18173		0.18173
$\rho_{14}$						0.81827		0.81827
$N_{dec}$	1	1	15	15	1	1	5	5
${\left(\frac{E_{b}}{N_{0}}\right)}_{dB}^{*}$	1.67	1.45	1.67	1.45	3.40	3.32	3.40	3.32
$\Gamma_{dB}^{*}$	1.35				3.12			
${\left(\frac{E_{b}}{N_{0}}\right)}_{dB}^{*}-\Gamma_{dB}^{*}$	0.32	0.10	0.32	0.10	0.28	0.20	0.28	0.20

**Table 4:** Optimal parameters of LDPC codes and JDD strategy for a coded massive MIMO system with R=0.5 over 16×16 and 64×64 channels, where GIO JDD with $N_{g}^{GIO}=15$ was used in a reference system.

Channels		16×16								64×64							
JDD Strategy		GIO				Proposed				GIO				Proposed			
$d_{v,\max}$		3		24		12		24		3		24		12		24	
$\lambda_{2}$						0.30583		0.25299						0.30486		0.25777	
$\lambda_{3}$		1.0		0.98521		0.38338		0.33001		1.0		0.98401		0.37864		0.32276	
$\lambda_{9}$								0.06158								0.05931	
$\lambda_{12}$						0.31079								0.31650			
$\lambda_{24}$				0.01479				0.35542				0.01599				0.36016	
$\rho_{6}$		0.99762		0.90687		0.42250				0.98853		0.89048		0.40215			
$\rho_{7}$		0.00238		0.09313		0.57750		0.18756		0.01147		0.10952		0.59785		0.21765	
$\rho_{8}$								0.81244								0.78235	
$N_{dec}$	$N_{g}$	1	15	1	15	21	11	18	11	1	15	1	15	35	13	29	13
${\left(\frac{E_{b}}{N_{0}}\right)}_{dB}^{*}$		3.24		3.23		1.91		1.86		3.20		3.19		1.82		1.69	
$\Gamma_{dB}^{*}$		1.35								1.35							
${\left(\frac{E_{b}}{N_{0}}\right)}_{dB}^{*}-\Gamma_{dB}^{*}$		1.89		1.88		0.56		0.51		1.85		1.84		0.47		0.34	

**Table 5:** Optimal parameters of LDPC codes and JDD strategy for a coded massive MIMO system with R=0.75 over 16×16 and 256×256 channels, where GIO JDD with $N_{g}^{GIO}=15$ was used in a reference system.

Channels		16×16						256×256					
JDD Strategy		GIO		Proposed				GIO		Proposed			
$d_{v,\max}$		3		3		20		3		3		20	
$\lambda_{2}$		0.00339		0.18128		0.15084		0.00772		0.18124		0.15360	
$\lambda_{3}$		0.99661		0.81872		0.65081		0.99228		0.81876		0.66624	
$\lambda_{20}$						0.19835						0.18017	
$\rho_{11}$				0.96429						0.96186			
$\rho_{12}$		1.0		0.03571				1.0		0.03814			
$\rho_{13}$						0.72658						0.97718	
$\rho_{14}$						0.27342						0.02282	
$N_{dec}$	$N_{g}$	1	15	11	13	9	13	1	15	76	14	67	14
${\left(\frac{E_{b}}{N_{0}}\right)}_{dB}^{*}$		4.20		3.66		3.65		4.10		3.45		3.44	
$\Gamma_{dB}^{*}$		3.12						3.12					
${\left(\frac{E_{b}}{N_{0}}\right)}_{dB}^{*}-\Gamma_{dB}^{*}$		1.08		0.54		0.53		0.98		0.33		0.32	
